# Extraocular motoneurons of the adult rat show higher levels of vascular endothelial growth factor and its receptor Flk-1 than other cranial motoneurons

**DOI:** 10.1371/journal.pone.0178616

**Published:** 2017-06-01

**Authors:** Silvia Silva-Hucha, Rosendo G. Hernández, Beatriz Benítez-Temiño, Ángel M. Pastor, Rosa R. de la Cruz, Sara Morcuende

**Affiliations:** Laboratorio de Fisiología y Plasticidad Neuronal, Departamento de Fisiología, Facultad de Biología, Universidad de Sevilla, Sevilla, Spain; Szegedi Tudomanyegyetem, HUNGARY

## Abstract

Recent studies show a relationship between the deficit of vascular endothelial growth factor (VEGF) and motoneuronal degeneration, such as that occurring in amyotrophic lateral sclerosis (ALS). VEGF delivery protects motoneurons from cell death and delayed neurodegeneration in animal models of ALS. Strikingly, extraocular motoneurons show lesser vulnerability to neurodegeneration in ALS compared to other cranial or spinal motoneurons. Therefore, the present study investigates possible differences in VEGF and its main receptor VEGFR-2 or Flk-1 between extraocular and non-extraocular brainstem motoneurons. We performed immunohistochemistry and Western blot to determine the presence of VEGF and Flk-1 in rat motoneurons located in the three extraocular motor nuclei (abducens, trochlear and oculomotor) and to compare it to that observed in two other brainstem nuclei (hypoglossal and facial) that are vulnerable to degeneration. Extraocular motoneurons presented higher amounts of VEGF and its receptor Flk-1 than other brainstem motoneurons, and thus these molecules could be participating in their higher resistance to neurodegeneration. In conclusion, we hypothesize that differences in VEGF availability and signaling could be a contributing factor to the different susceptibility of extraocular motoneurons, when compared with other motoneurons, in neurodegenerative diseases.

## Introduction

Amyotrophic lateral sclerosis (ALS) is a neurodegenerative disease characterized by a progressive loss of upper and lower motoneurons in the central nervous system. However, not all motoneurons are equally vulnerable to the disease. Whilst most motoneuron pools are damaged, motoneurons of the oculomotor system are resistant to neurodegeneration in ALS. At early stages of the disease, a severe neuronal loss is consistently observed in the facial, trigeminal and hypoglossal nuclei, producing profound deficits of tongue motility, dysphagia and dysarthria [1,2]. By contrast, the oculomotor, trochlear and abducens nuclei remain unaffected in human ALS and in animal models of ALS [1–4]. Unraveling the characteristics that distinguish vulnerable from non-vulnerable motoneurons in ALS disease will allow a better understanding of the mechanisms involved in neurodegeneration and, therefore, the development of new therapeutic strategies.

Several aspects distinguish extraocular muscle motoneurons from other brainstem motoneurons. Thus, extraocular motoneurons are enriched in calcium buffering proteins, mainly parvalbumin [2,5,6], which could provide them with a higher resistance to neurodegeneration due to disruption of calcium homeostasis observed in ALS disease [7,8]. On the other hand, previous studies have revealed that the relative abundance of the neuropeptide calcitonin gene-related peptide (CGRP) in motoneurons predicts their vulnerability in ALS models, thus suggesting that CGRP might be an autocrine or paracrine factor promoting motoneuron degeneration. Motoneurons of the extraocular system show low expression of CGRP when compared to other vulnerable motoneurons, like facial or spinal ones [9,10]. Another characteristic that differentiates extraocular motoneurons from others is the presence of the specific receptor for nerve growth factor (NGF), TrkA, in the adult animal [10–12]. Other cranial or spinal motoneurons express the high affinity receptor for brain-derived neurotrophic factor (BDNF), TrkB, or neurotrophin-3 (NT-3), TrkC, in the adult, but lack TrkA [13–16]. Extraocular motoneurons also express TrkB and TrkC [11]. Thus, the specific neurotrophic action of NGF in ocular motoneurons could be also contributing to their higher resistance to neurodegeneration. However, clinical trials of neurotrophins application to patients led to variable results and they did not reach the expected success [17].

The vascular endothelial growth factor (VEGF) has been proposed as a neurotrophic and neuroprotective factor for motoneurons, since a decrease in VEGF supply has been related to motoneuron degeneration in motor system diseases such as ALS [18,19]. VEGF has been classically a well-known angiogenic factor, but the fact that impairing VEGF production through genetic manipulation results in degeneration of motoneurons, resembling ALS [20], has revealed that the ability to produce endogenous VEGF may be important for maintaining the health of motoneurons in the brainstem and spinal cord. In addition, VEGF delivery through retrograde transport of a lentiviral vector injected into several muscles delays onset and prolongs the survival of SOD1^G93A^ mice, an animal model of ALS [21], and its intracerebroventricular infusion preserves neuromuscular junction in the SOD1^G93A^ rat model of ALS [22]. These findings have increased the interest in evaluating the therapeutic potential of VEGF for neurodegenerative disorders. VEGF is known to mediate its biological functions via activation of the protein tyrosine kinase receptors, VEGF receptor 1 (VEGFR-1/Flt-1) and VEGFR-2 (KDR/Flk-1), although most evidence points to Flk-1 as the main receptor mediating VEGF action in neuronal function [23,24].

Therefore, it would be interesting to explore whether the lesser vulnerability to degeneration observed in extraocular motoneurons could be, at least in part, due to a higher amount of VEGF. In order to achieve this goal, the presence of VEGF and its receptor Flk-1 was analyzed by immunohistochemistry and Western blot in the extraocular motor nuclei, and compared with that observed in other non-extraocular cranial motoneurons located in the facial and hypoglossal nuclei, which are susceptible to neurodegeneration [3,4].

## Materials and methods

Experiments were performed in adult Wistar rats in accordance with the guidelines of the European Union (2010/63/EU) and Spanish law (R.D. 53/2013, BOE 34/11370-421) for the use and care of laboratory animals. Surgical procedures used in this study were approved by the ethics committee of Universidad de Sevilla.

### Immunohistochemistry

Adult rats (n = 9) were perfused transcardially under deep anesthesia (sodium pentobarbital, 50 mg/kg, i.p.) with 100 ml of physiological saline followed by 250 ml of 4% paraformaldehyde in 0.1 M sodium phosphate buffer, pH 7.4 (PB). The brainstem was removed and cryoprotected by immersion in a solution of 30% sucrose in sodium phosphate-buffered saline (PBS) until sinking. Tissue was then cut coronally in 50-μm-thick sections using a cryostat (Leica CM1850, Wetzlar, Germany). Brainstem sections were divided in two series: one for VEGF immunohistochemistry, and the other for Flk-1, selecting alternate sections for each series.

After washing in PBS, coronal sections containing the oculomotor, trochlear, abducens, facial and hypoglossal nuclei were incubated in 1% sodium borohydride prepared in PBS for 10 minutes for antigen retrieval, and then rinsed again in PBS. Double immunofluorescence was made for choline acetyltransferase (ChAT), the biosynthetic enzyme of acetylcholine, therefore a general marker for motoneurons, that was used in this work to identify the brainstem motor nuclei, along with either VEGF or Flk-1, using TRICT, Cy2 or FITC as fluorophores, respectively.

For VEGF immunohistochemistry, nonspecific binding was blocked by incubation for 45 minutes in a solution consisting of 10% normal horse serum in PBS with 0.01% Triton X-100 (PBS-T). Sections were then incubated overnight at room temperature with the primary antibody solution containing rabbit polyclonal anti-VEGF IgG (Santa Cruz Biotechnology, Dallas, TX, USA; sc-507, 1:100) prepared in PBS-T with 5% of normal horse serum. After several rinses in PBS-T, sections were incubated for 2 hours in a biotinylated secondary antibody in PBS-T (biotinylated horse anti-rabbit IgG; Vector Labs, Burlingame, CA, USA; BA-110, 1:500), washed again and finally transferred for 45 minutes to a solution containing streptavidin-Cy2 (Jackson ImmunoResearch, West Grove, PA, USA; 016-220-084, 1:400).

In alternate sections of the same animals, immunohistochemistry against Flk-1 was performed. Sections were incubated for 45 minutes in a blocking solution consisting of 10% normal donkey serum in PBS-T, and then they were incubated for 3 hours at room temperature with the primary antibody solution containing mouse monoclonal anti-Flk-1 IgG (Santa Cruz Biotechnology; sc-6251, 1:1000) prepared in PBS-T with 5% of normal donkey serum. After several rinses in PBS-T, sections were incubated for 2 hours in the secondary antibody solution (donkey anti-mouse-FITC IgG; Jackson ImmunoResearch; 715-095-150, 1:50).

In order to identify cranial motoneurons in the brainstem, subsequent immunohistochemistry for the detection of ChAT was performed in all sections. The primary antibody was a goat polyclonal anti-ChAT IgG from Millipore (Billerica, MA, USA; AB-144P, 1:500) and the antibody binding was visualized by incubating tissue with an anti-goat-TRITC IgG (Jackson ImmunoResearch; 705-025-003, 1:50).

Sections were then washed for several times, mounted on glass slides and coverslipped with a 0.1 M solution of n-propyl gallate prepared in glycerol:PBS (9:1).

### Antibody characterization

**Fig 1:**
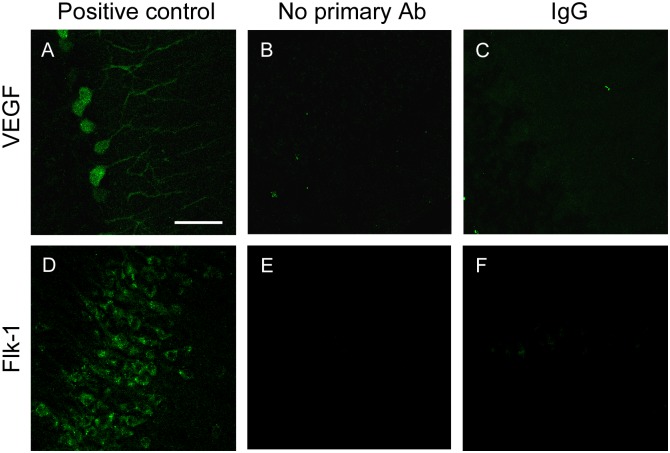
Antibody characterization. Confocal microscopy images of rat brain sections through the cerebellum (A-C) or hyppocampus (D-F) showing immunoreactivity to VEGF and Flk-1 in positive controls (A and D) and negative controls consisting in the omission of the primary antibody (Ab; B and E), or the tissue pre-incubation with normal rabbit IgG instead of VEGF (C) and normal mouse IgG instead Flk-1 (F) antibodies. Scale bar: 50 μm.

For positive controls, the immunohistochemistry protocol was performed in brain sections containing cerebellum and hippocampus, areas previously reported as immunopositive for VEGF and Flk-1, respectively [23,25,26]. As previously described, Purkinje cells in the cerebellum were immunopositive for VEGF (Fig 1A), meanwhile granular cells in the hippocampus were immunopositive for Flk-1 (Fig 1D). No blocking peptide for VEGF or Flk-1 was available by provider. Then negative controls were carried out by omission of the primary antibodies, resulting in absence of staining in all cases (Fig 1B and 1E). Additional negative controls were performed after replacing the primary antibody incubation by normal rabbit IgG (Jackson Immunoresearch; 011-000-002) or normal mouse IgG (Jackson Immunoresearch; 015-000-002) at the same concentration than the primary antibody. Again, no specific labeling was found (Fig 1C and 1F). We have previously described the specificity of ChAT [27] and VEGF [26] antibodies. The antibody used against Flk-1 receptor selectively detects the presence of this receptor in neurons, but not in endothelial cells [28].

### Confocal microscopy and optical density quantification

In order to analyze differences between brainstem motor nuclei in the number of VEGF- or Flk-1-immunostained motoneurons, confocal microscopy images of motoneurons were captured at 40X magnification with a confocal laser-scanning microscope (Zeiss LSM 7 DUO, Oberkochen, Germany). Different focal planes containing the cell nucleus were scanned by exciting the FITC or Cy2 fluorophores with 488-nm argon laser and the TRITC with 543-nm laser. Signal intensity was measured by outlining the soma of individual motoneurons (avoiding the cell nucleus) on images of both sides in every section and calculating the average pixel intensity (i.e., the optical density) using ImageJ software (NIH) [26]. In order to avoid any bias against motoneurons with low levels of VEGF or Flk-1 expression, all motoneurons (identified by ChAT staining) within the focal plane of our confocal images (identified by a clear nucleus within the soma) were included in the analysis. For background correction, four optical density readings were taken per image in areas devoid of motoneurons [29]. Then, the optical density value of every cell was divided by the mean background level determined for the same image. Motoneurons were considered as immunopositive for VEGF or Flk-1 when they showed an optical density value at least three times higher than the background level. Data are presented as the mean percentage of VEGF- or Flk-1-positive motoneurons (identified by their ChAT labeling) per nucleus obtained in the different animals (n = 9).

### Western blot analysis

Adult rats (n = 12) were decapitated under deep anesthesia, and their brains were removed. Under microscopic observation, the oculomotor complex (including trochlear and oculomotor nuclei [3]), the facial and the hypoglossal nuclei were dissected. The tissue was homogenized in cold lysis buffer containing a cocktail of protease and phosphatase inhibitors [30], disrupted by sonication and centrifuged at 13000 rpm for 30 minutes. The supernatants were isolated and total protein concentrations were determined by the Bradford method [31], using BSA as a standard. Proteins were diluted in sample buffer and denatured at 95°C for 6 minutes, and they were separated by 15% (VEGF) or 7.5% (Flk-1) SDS PAGE (50 μg/lane) and transferred to PVDF membrane by electroblotting. To reduce nonspecific binding, the membranes were blocked for 1 hour with 10% BSA, and then blots were incubated overnight at 4°C in a solution containing anti-VEGF rabbit polyclonal antibody (Abcam, Cambridge, MA, USA; ab46154, 1:1000) or anti-Flk-1 rabbit polyclonal antibody (Abcam, Cambridge, MA, USA; ab11939, 1:1000), diluted in TBS-Tween 0.1% supplied with 5% BSA. After washing three times with PBS-Tween buffer, the membranes were incubated with horseradish peroxidase-conjugated anti-rabbit antibody (Vector Labs; PI-1000, 1:200) 1 hour at room temperature. The immunoreaction was detected using the WesternBright Quantum kit (Advansta, Menlo Park, CA, USA; K-12042). The chemiluminescence was visualized using a Luminescent Image Analyzer (LAS-3000, Fuji Photo Film GmbH, Düsseldorf, Germany). After washing the membranes for 10 minutes with stripping buffer, blots were reprobed with anti-glyceraldehyde 3-phosphate dehydrogenase (GAPDH) mouse monoclonal antibody (Millipore; MAB374, 1:1000) to ensure equal loading. The density of the immunoreactive bands was quantified by densitometry using the Multi Gauge software (Fuji Photo Film, Japan). The data were normalized to the GAPDH level for each sample. VEGF or Flk-1 expression in facial and hypoglossal nuclei was expressed relative to that found in the oculomotor complex for each Western blot. For positive controls of the antibodies, proteins extractions from rat cerebellum and hippocampus were included as positive controls of VEGF and Flk-1 antibodies, respectively.

### Statistics

The percentage of VEGF-positive or Flk-1-positive motoneurons per nucleus was compared between nuclei for the immunostaining experiments. The expression of VEGF and Flk-1 proteins obtained from the Western blots in the oculomotor complex, facial and hypoglossal nuclei was normalized to the GAPDH level expression, and then the expression level in the facial and hypoglossal nuclei was indicated relative to the oculomotor complex. Comparisons between nuclei were made with the program SigmaPlot 11 (Systat Software GmbH, Erkrath, Germany) by using the one-way ANOVA test followed by *post hoc* multiple comparisons (Holm-Sidak method) at a significance level of p < 0.05. Data were expressed as mean ± standard error of the mean (SEM).

## Results

The major aim of the present work has been to determine whether the lower vulnerability to degeneration observed in extraocular motoneurons [2,3,5,29,32], when compared to other cranial motoneurons, could be explained by a higher trophic support by VEGF, according to recent evidences linking the deficit in this factor with motoneuron disease [20]. For this purpose, we explored and quantitatively compared the presence of VEGF and the expression of its receptor Flk-1 between different populations of cranial motoneurons, by means of immunohistochemistry at the confocal microscopy level and Western blot analysis.

### Presence of VEGF in brainstem motoneurons

**Fig 2:**
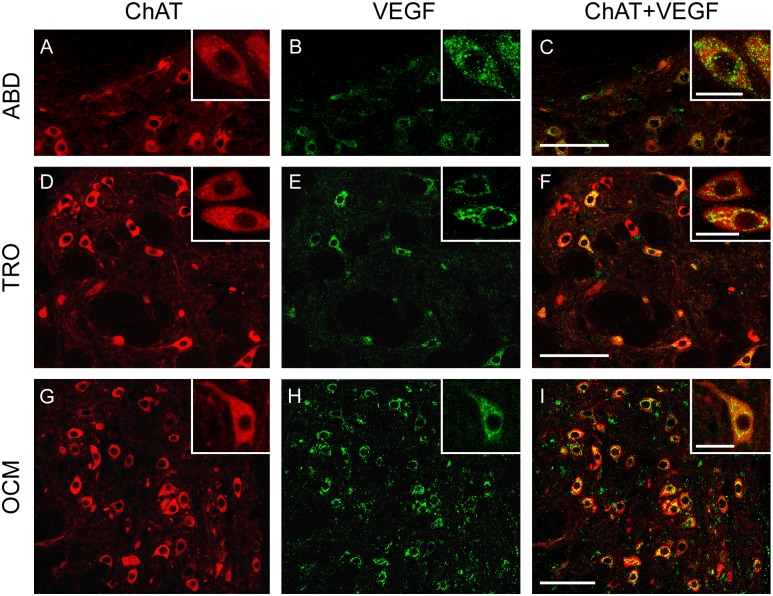
VEGF immunoreactivity in extraocular motoneurons. Confocal microscopy images showing the presence of VEGF in the motoneurons of the extraocular system of the adult rat (ABD: abducens nucleus; TRO: trochlear nucleus; OCM: oculomotor nucleus). Extraocular motoneurons, identified by ChAT immunostaining (in red; A, D and G), showed intense immunoreactivity to VEGF (in green; B, E and H). The right column shows the merged images. Insets illustrate higher magnification images of doubly-labeled motoneurons in every nucleus. Scale bars = 100 μm (in C for A-C; in F for D-F; in I for G-I). Inset scale bars = 25 μm.

In order to study and quantify the presence of VEGF in brainstem motoneurons, a double immunohistochemistry protocol was performed. Motoneurons were identified by their positive immunostaining against ChAT (red color, TRITC) (Fig 2A, 2D and 2G). In addition, VEGF immunolabeling (in green, Cy2) was also used revealing the presence of this factor in the soma of extraocular motoneurons, which were located in the abducens, trochlear and oculomotor nuclei (Fig 2B, 2E and 2H; respectively). The overlay of ChAT and VEGF images revealed the colocalization of both proteins in the majority of extraocular motoneurons (Fig 2C, 2F and 2I), showing their strong immunoreactivity for VEGF.

**Fig 3:**
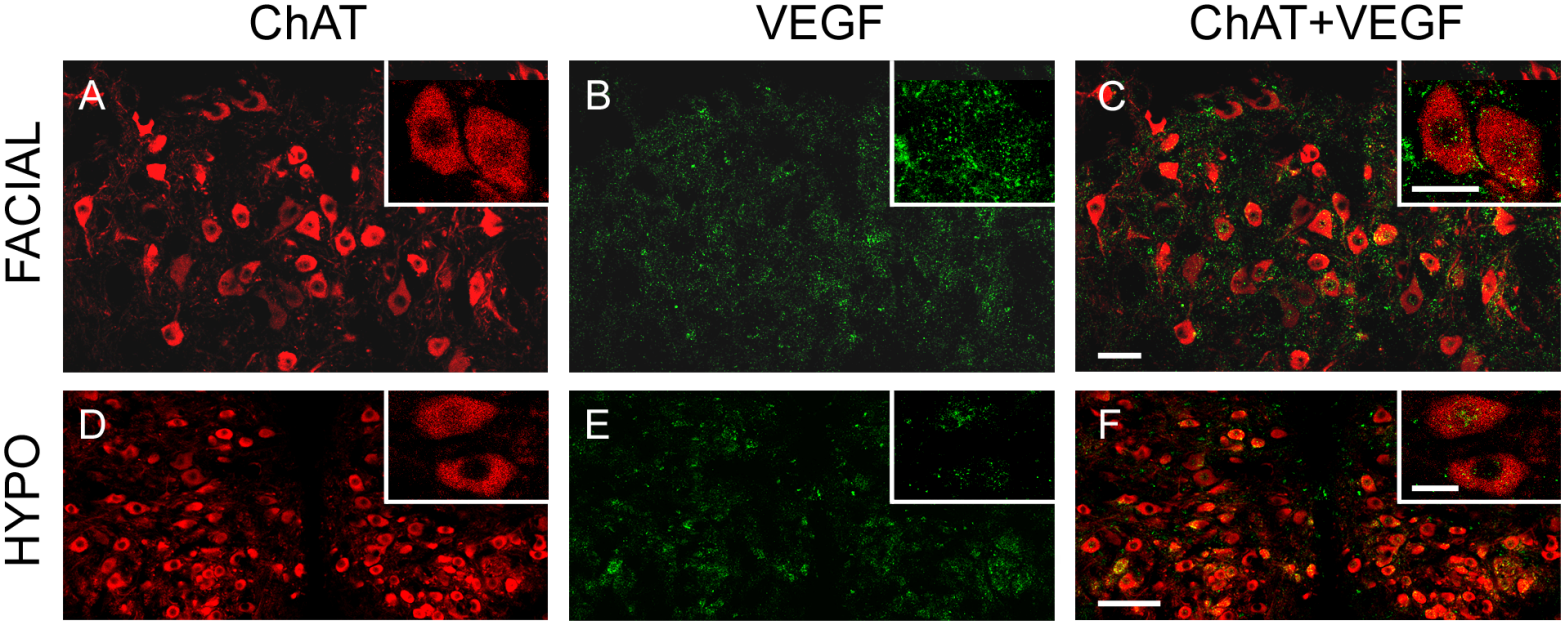
VEGF immunoreactivity in facial and hypoglossal motoneurons. Representative confocal microscopy images of facial and hypoglossal (HYPO) motoneurons, immunopositive for ChAT (in red; A and D, respectively), showing weak immunoreactivity to VEGF (in green; B and E, respectively). The right column shows the merged images. Insets illustrate higher magnification images of motoneurons. Scale bars = 100 μm (in C for A-C; in F for D-F). Inset scale bars = 25 μm.

In contrast, sections from the same animals showed a faint VEGF immunolabeling in other cranial motoneurons, like those involved in the control of facial muscles and the tongue, located in the facial and hypoglossal nuclei, respectively. Fig 3 shows ChAT-immunopositive facial (Fig 3A) and hypoglossal (Fig 3D) motoneurons whose level of VEGF immunostaining was very low or absent (Fig 3B and 3E, respectively). As a consequence, the merge of ChAT and VEGF images yielded very few doubly-labeled motoneurons (Fig 3C and 3F).

### VEGF immunoreactivity is higher in extraocular motoneurons than in other cranial motoneurons

The intensity of VEGF immunolabeling was evaluated by quantifying the optical density within the motoneuron soma in every motor nucleus included in the present study (abducens, trochlear, oculomotor, facial and hypoglossal nuclei). We considered a cell positive for VEGF when its optical density was at least three times higher than the background signal (see Materials and methods for details).

**Fig 4:**
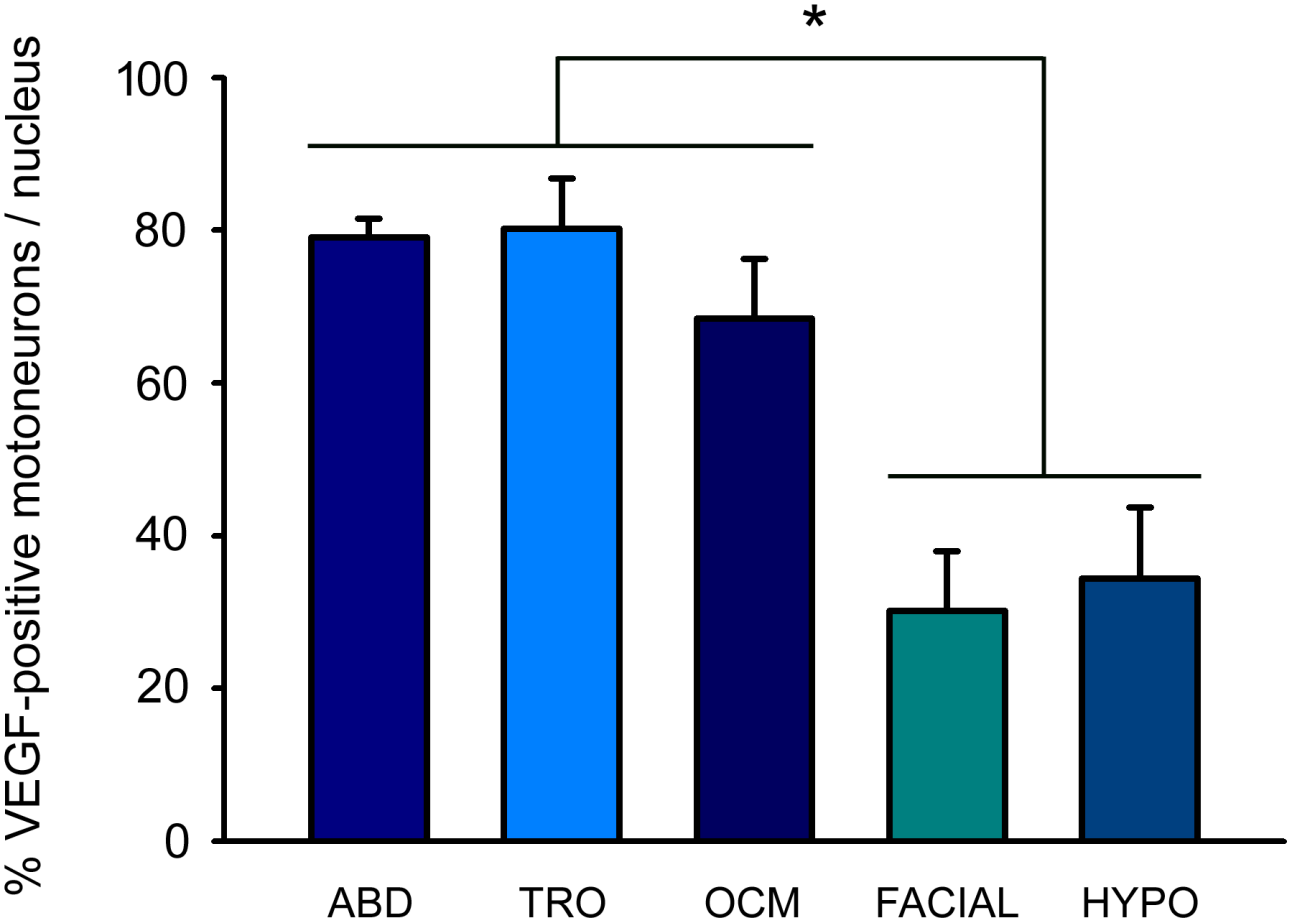
VEGF immunoreactivity is higher in extraocular motoneurons than in non-extraocular brainstem motoneurons. Quantification of the percentage of VEGF-positive motoneurons in cranial motor nuclei. Note the higher percentage of motoneurons positive for this factor in the ocular motor system, in contrast to the low number observed in the motoneurons of the facial or lingual motor systems. Data represent mean ± SEM. The asterisk represents significant differences between groups (one-way ANOVA test followed by Holm-Sidak method for multiple pairwise comparisons; *, p < 0.001; n = 9 animals).

When the percentage of VEGF-immunopositive motoneurons was calculated in each motor group, we found that the majority of motoneurons within the extraocular motor nuclei were immunopositive for VEGF (abducens: 79.11 ± 2.44%; trochlear: 80.26 ± 6.53%; oculomotor: 68.43 ± 7.87%), and that there were no statistically significant differences between these three extraocular nuclei (Fig 4; n = 9 animals).

However, significant differences (p < 0.001; one-way ANOVA test followed by *post hoc* Holm-Sidak method) were obtained when extraocular motor nuclei were compared to facial and hypoglossal nuclei (Fig 4). The percentage of VEGF-immunoreactive motoneurons was around 30% and similar in both non-extraocular nuclei (facial: 30.18 ± 7.86%; hypoglossal: 34.44 ± 9.30%), with absence of significant differences between both nuclei.

Taken together, these results showed that the level of VEGF in motoneurons of the ocular motor system was higher than that present in other cranial motoneurons of the adult rat.

### Expression of Flk-1 in cranial motoneurons

To evaluate whether the differences observed in VEGF correlated with differences in the expression of the VEGF receptor Flk-1, we studied the presence of this receptor in the same motor pools. Double immunofluorescence against ChAT and Flk-1 was performed in the five cranial motor nuclei previously mentioned and results were analyzed at the confocal microscopy level.

**Fig 5:**
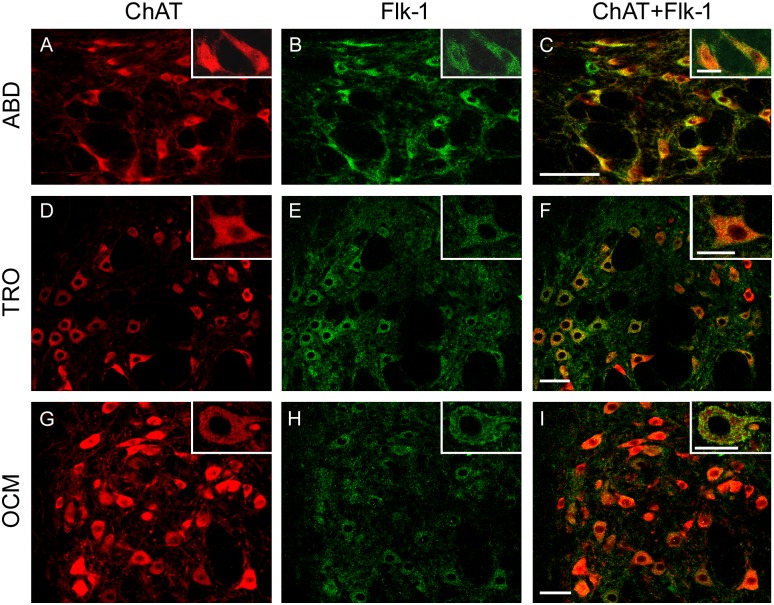
Flk-1 receptor is extensively distributed in extraocular motor nuclei. The left panel shows confocal images of the abducens (ABD), trochlear (TRO) and oculomotor (OCM) nuclei, whose motoneurons were identified by means of ChAT immunolabeling (TRICT, red), and expressed high levels of Flk-1 receptor (FITC, green; second column). The third column represents the overlay of ChAT and Flk-1 images. Insets show representative motoneurons of each nucleus at higher magnification. Scale bars = 100 μm (in C for A-C; in F for D-F; in I for G-I). Inset scale bars = 25 μm.

In the extraocular motor nuclei (i.e., abducens, trochlear and oculomotor), the vast majority of motoneurons, identified as ChAT-immunoreactive (Fig 5A, 5D, and 5G) also exhibited Flk-1 immunostaining (Fig 5B, 5E and 5H), as shown in the merged images (Fig 5C, 5F and 5I).

**Fig 6:**
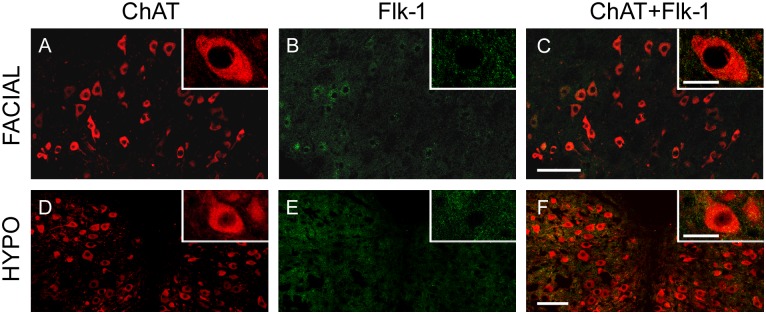
Facial and hypoglossal motoneurons express low levels of Flk-1 receptor. Confocal microscopy images of facial and hypoglossal (HYPO) motoneurons (A, D; identified as ChAT-immunopositive) showing low levels of Flk-1 immunoreactivity (B, E). C and F represent the merge of VEGF and Flk-1 images. Insets show motoneurons of each nucleus at higher magnification. Scale bars = 100 μm (in C for A-C; in F for D-F). Inset scale bars = 25 μm.

However, the presence of this receptor in facial and hypoglossal motoneurons was much less noticeable. Only a faint immunolabeling against Flk-1 was detectable in a reduced number of facial and hypoglossal motoneurons, as shown in Fig 6.

**Fig 7:**
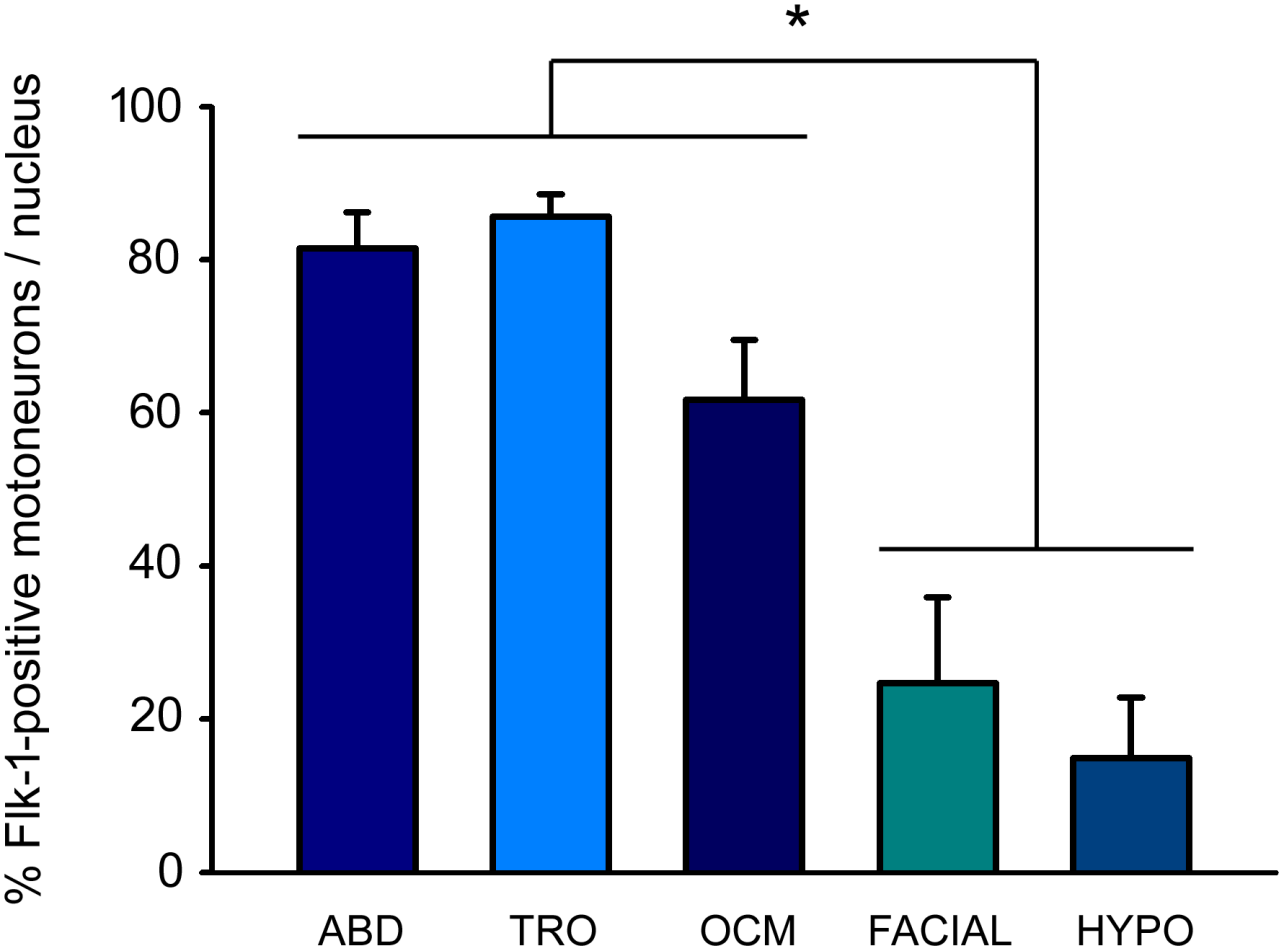
Extraocular motoneurons express higher levels of Flk-1 receptor than non-extraocular brainstem motoneurons. The bar chart shows the mean and SEM of the percentage of motoneurons expressing Flk-1 receptor in each brainstem nucleus. The three extraocular motor nuclei showed significantly higher percentages of Flk-1-immunopositive motoneurons than the facial or hypoglossal nuclei. Data represent mean ± SEM. The asterisks represent significant differences between groups (one-way ANOVA test followed by Holm-Sidak method for multiple pairwise comparisons; *, p < 0.001; n = 9 animals).

The analysis of the optical density of Flk-1 in the extraocular motoneurons revealed a high proportion of motoneurons positive for the receptor (Fig 7). The percentage of Flk-1-positive neurons in the extraocular nuclei was 81.46 ± 4.79%, 85.68 ± 2.88%, and 61.72 ± 7.86% for the abducens, trochlear and oculomotor nucleus, respectively. Again, there were no significant differences between these three nuclei regarding Flk-1 presence.

In contrast, the percentage of motoneurons that were Flk-1-positive was much lower in the non-extraocular nuclei. In this case, only 24.72 ± 11.20% and 15.01 ± 7.87% of the motoneurons in the facial and hypoglossal nuclei were Flk-1-immunopositive, respectively. The difference in the percentage of Flk-1-immunoreactive motoneurons between extraocular motor nuclei as compared to facial and hypoglossal nuclei was found significant (one-way ANOVA followed by *post hoc* Holm-Sidak method; p < 0.001; n = 9 animals; Fig 7). On the other hand, the percentages obtained from the two latter groups were similar.

Therefore, these findings clearly indicated that the majority of extraocular motoneurons might be receptive to the molecule VEGF acting through its membrane receptor Flk-1, whereas other cranial motoneurons of the adult rat scarcely expressed this receptor and, in comparison, were less responsive to VEGF.

### Western blot analysis of VEGF and Flk-1 in cranial motor nuclei

We used Western blotting as a quantitative method to compare the total VEGF and Flk-1 proteins present in homogenized tissue obtained from the different brainstem motor nuclei.

**Fig 8:**
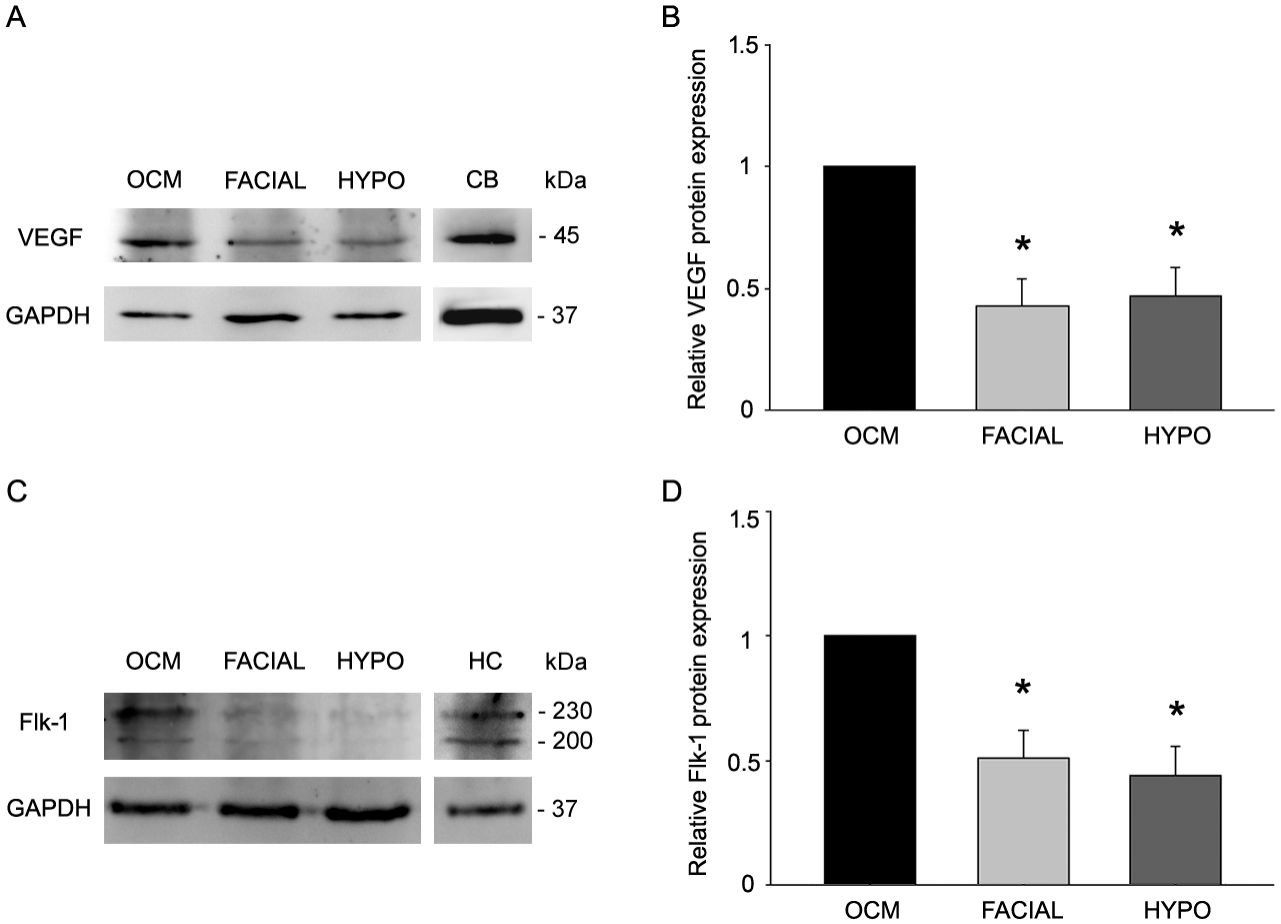
Western blot analysis of VEGF and Flk-1 proteins in rat cranial nuclei. A. The protein band for VEGF is shown for the oculomotor (OCM), facial and hypoglossal (HYPO) nuclei of the rat. Cerebellum (CB) protein extract was included as a positive control for VEGF protein. GAPDH immunoblotting was used as load control. B. Densitometry data showed a significantly lower amount of VEGF protein in facial and hypoglossal nuclei as compared with the oculomotor complex (one-way ANOVA test followed by Holm-Sidak method for multiple pairwise comparisons; differences with respect to OCM: *, p<0.001; n = 6 animals). C. Western blot for Flk-1 in oculomotor, facial and hypoglossal nuclei of the rat. Hippocampus (HC) protein extract was included as a positive control for Flk-1 protein. GAPDH immunoblotting was used as load control. D. A significantly higher amount of Flk-1 protein was quantified in the oculomotor complex as compared with facial and hypoglossal nuclei (one-way ANOVA test followed by Holm-Sidak method for multiple pairwise comparisons; differences with respect to OCM: *, p<0.001; n = 6 animals).

For VEGF, in all cases, one protein band was detected at the molecular weight of 45 kDa (Fig 8A), corresponding to the VEGF protein [33]. As for the immunocytochemistry, we also used cerebellum as a positive control for VEGF in the Western blots, obtaining a dense band at 45 kDa (Fig 8A). Densitometric analysis revealed that the basal VEGF amount was significantly higher in the oculomotor complex (i.e., the oculomotor and trochlear nuclei together) compared with facial or hypoglossal nuclei and similar between these two latter motor nuclei (normalized value of 1 for the oculomotor complex; 0.42 ± 0.12 and 0.47 ± 0.12 for the facial and hypoglossal nuclei, respectively; p < 0.001; one-way ANOVA test followed by *post hoc* Holm-Sidak method) (Fig 8B; n = 6 animals). Therefore, the results of Western blots were consistent with those obtained from the immunohistochemical study.

The Western blot for Flk-1 showed the presence of two bands of 200 and 230 kDa of molecular weight, the latter corresponding to the mature form of Flk-1 protein [34,35], and therefore being the one to be analyzed (Fig 8C). As a positive control for Flk-1, the Western blotting of the hippocampus revealed a clear band at this molecular weight (Fig 8C). The results of the analysis showed that Flk-1 protein level was significantly lower in facial (0.51 ± 0.11) or hypoglossal (0.44 ± 0.12) nuclei compared with the oculomotor complex (normalized value of 1 for the oculomotor complex; p < 0.001; one-way ANOVA test followed by *post hoc* Holm-Sidak method) (Fig 8D; n = 6 animals). No differences were observed in the level of Flk-1 protein between facial and hypoglossal nuclei. Thus, these results supported those obtained by immunohistochemistry.

## Discussion

The main purpose of this work has been to test whether there is a difference in the availability of the neurotrophic factor VEGF for motoneurons of the oculomotor system, which are known to be resistant to degeneration, compared to other cranial motoneurons whose resistance is lower. Therefore, we have quantified the presence of VEGF and its receptor Flk-1 in the motor nuclei containing extraocular motoneurons, and have compared the results with those obtained from other brainstem motor nuclei containing the motoneurons that control facial and tongue muscles, which are vulnerable to degeneration.

### Presence of VEGF in motoneurons of the oculomotor, facial and hypoglossal systems

One of the major findings of the present study is that the percentage of adult rat motoneurons containing the neurotrophic factor VEGF, known to enhance motoneuronal survival [21,22,36–39], is higher in extraocular motor nuclei than in other brainstem motor nuclei, in particular the facial and hypoglossal nuclei. The immunohistochemical study revealed that the majority of the extraocular motoneurons were positive for VEGF labeling, whereas only few facial and hypoglossal motoneurons showed VEGF immunoreactivity. Western blot analysis further corroborated these findings showing that the oculomotor complex presented quantitatively higher amounts of total VEGF protein in comparison to facial or hypoglossal nuclei. Therefore, our data suggest the possibility that the differential VEGF content might be involved in the dissimilar response of these two groups of brainstem motoneurons to neurodegeneration in motoneuronal maladies, such as ALS.

Previous studies have also reported a weak VEGF immunoreactivity in hypoglossal and facial motoneurons in control rats, highlighting the importance of VEGF presence in the soma for maintaining the health of motoneurons in the brainstem and spinal cord [18]. It is still unknown whether the high content of VEGF in the soma of extraocular motoneurons is due to endogenous synthesis (if so, VEGF would be acting autocrinally), or alternatively VEGF might reach the motoneurons via either paracrine pathways (e.g., from nearby glial cells), or retrograde axonal transport from the target muscles, or even anterogradelly from their afferences.

In neurodegenerative diseases there is a selective preservation of certain brainstem motoneuron pools such as the oculomotor/trochlear, as opposed to the trigeminal, facial and hypoglossal nuclei which are involved early in the course of the disease [3,4]. Several hypotheses have been proposed to explain the differential vulnerability of these motoneuronal populations. Thus, in contrast to facial and hypoglossal motoneurons, which have relatively low calcium buffering capacity, the oculomotor system is characterized by its wealth in calcium-binding proteins, particularly parvalbumin [6]. This fact, together with a differential expression of glutamate receptors between these motoneurons [40,41], has been proposed to prevent motoneuronal degeneration caused by glutamate overstimulation [2,5].

In fact, one of the main mechanisms that have been related to the neurodegeneration associated to ALS pathogenesis is glutamate excitotoxicity. Motoneurons are particularly susceptible to excitotoxicity because they present a high number of Ca^2+^-permeable AMPA receptors [42], compared to other types of neurons. Only AMPA receptors lacking the GluR2 subunit are permeable to Ca^2+^ ions, and motoneurons are relatively deficient in GluR2 [43]; consequently, they have a higher risk of Ca^2+^ entry. Calcium ions induce the generation of reactive oxygen species in mitochondria, which damage the motoneurons in which they are produced [44].

It has been described that VEGF protects motoneurons against excitotoxicity by up-regulating the transcription of GluR2 subunit [24]. Thus, if extraocular motoneurons express higher amounts of VEGF, this trophic factor could be contributing to their survival by increasing the incorporation of GluR2 subunit in the AMPA receptor complex, protecting them from excitotoxicity. This could be one of the reasons of the lesser vulnerability to neurodegeneration presented by these specific motoneuron pools compared to other types of motoneuron. Therefore, the fact that extraocular motoneurons are endowed with higher amounts of VEGF (present results) could be related to their higher resistance to cell death when they are exposed to an excitotoxic environment, as has been proposed to occur in ALS [45]. Indeed, several reports from other investigators have provided evidences about the reduced susceptibility to excitotoxicity showed by extraocular motoneurons [32,40,46], suggesting that it could be an important determinant of their resistance to degeneration.

VEGF expression increases in diverse types of neurons (e.g., cortical and hippocampal) in response to several insults, such as ischemia and seizures, and it has been suggested that VEGF up-regulation could be involved in the protection against neuronal loss [47,48]. Indeed, low levels of VEGF has been detected in the cerebrospinal fluid and in the anterior horn cells of the spinal cord in ALS patients [33,49,50]. Furthermore, impairing VEGF production through genetic manipulation results in degeneration of lower motoneurons in the adulthood [20]. It is important to highlight that our results are consistent with the neuroprotective role of VEGF. This neurotrophic factor has been proven to have a great therapeutic potential prolonging motoneuron survival and enhancing motor performance [21,36,50,51]. Accordingly, it represents a promising molecule for ALS treatment.

ALS pathogenesis is not confined only to motoneurons, but rather increasing evidences suggest that surrounding non-neuronal cells are also affected and, indeed, they might play an important role in the alterations observed in motoneurons. Glia is known to express VEGF receptors and to modulate synaptic transmission [52,53]. Astrocytes have been reported to modulate the vulnerability of motoneurons to excitotoxicity, influencing the GluR2 expression in spinal motoneurons [54]. In fact, as VEGF protects motoneurons against excitotoxicity by up-regulating GluR2 expression [24], then astrocytes could be contributing to the higher resistance of extraocular motoneurons by providing them with VEGF.

Recently, intrathecal delivery of VEGF has been reported to increase the survival of spinal motoneurons in SOD1^G93A^ mice by activation of the phosphatidyl-inositol-3-kinase (PI3K) /Akt survival pathway and increasing the level of the anti-apoptotic factor Bcl-2, but also through the induction of a switch in the microglial phenotype from M1, the pro-inflammatory state, to M2, the neuroprotective one [55]. Therefore, the higher levels of VEGF shown by the oculomotor system could be promoting the anti-inflammatory state of the microglial cells surrounding extraocular motoneurons, although this hypothesis has not been proven yet.

### Expression of Flk-1 in motoneurons of the oculomotor, facial and hypoglossal systems

Another important finding of the present work has been to show that a higher percentage of extraocular motoneurons express Flk-1 when compared with other brainstem motor pools (i.e., facial and hypoglossal). A higher amount of Flk-1 in oculomotor nuclei was also quantified by Western blot, in comparison to facial or hypoglossal nuclei. Although stimulation of VEGFR-1 (the other tyrosine kinase receptor of VEGF) has also been reported to protect neurons in various models of neuronal death [56,57], the neuroprotective properties of VEGF are mostly mediated by Flk-1 [20,24,58–60]. Ligand binding to Flk-1 leads to autophosphorylation and subsequent activation of intracellular pathways, including PI3-K, phospholipase C, and mitogen-activated protein kinase (MEK) [61], whose activation are known to mediate the protection induced by VEGF against motoneuron death. Furthermore, Flk-1 blockade prevents the VEGF-mediated protection against motoneuron death both *in vivo* [39,62,63] and *in vitro* [58]. In addition, low levels of this receptor and its transcript have been detected in anterior horn cells of the spinal cord of ALS patients [33].

Altogether, it can be suggested that the high levels of Flk-1 expression in the motoneurons of the oculomotor system found in the present study might contribute to their relative resistance to degeneration.

## Conclusions

The presence of VEGF and its receptor Flk-1 in extraocular motoneurons suggests that VEGF could be acting as a neurotrophic factor for these cells. Moreover, the percentage of extraocular motoneurons containing VEGF or Flk-1, as well as the amount of these proteins, are significantly higher than in other brainstem motor pools (i.e., facial and hypoglossal). These differences in VEGF signaling between ocular and non-ocular motoneurons suggest that VEGF may be an important factor for motoneuronal survival and could contribute to the increased resistance observed in extraocular motoneurons to neurodegenerative diseases, such as ALS.
